# Diabetes Mellitus: An Unrecognized Complication in the Management of Patients With Mental Illness

**DOI:** 10.7759/cureus.8444

**Published:** 2020-06-04

**Authors:** Diana Espinoza, Paola A Sanchez, Christine Junia

**Affiliations:** 1 Internal Medicine, MacNeal Hospital, Berwyn, USA

**Keywords:** mental illness, antipsychotics, hhs, rhabdomyolysis, nms, aki, diabetes mellitus, high risk

## Abstract

Psychiatric patients can have undiagnosed illness due to difficulties accessing the health care system and the lack of guidelines regarding screening recommendations for them. The following case describes a 36-year-old male who presented with a hyperglycemic emergency in the setting of undiagnosed diabetes mellitus.

## Introduction

The prevalence of diabetes and other cardiovascular risk factors seems to be higher in psychiatric patients compared to the general population [1]. Type 2 diabetes mellitus (T2DM) often presents at an earlier age or with acute metabolic emergencies in this population. One possible contributor is the side effects of antipsychotics, which affect metabolism through various mechanisms and the challenge of a patient's psychiatric comorbidity contributing to poor access to preventive care and chronic disease management [2].

## Case presentation

**Figure 1:**
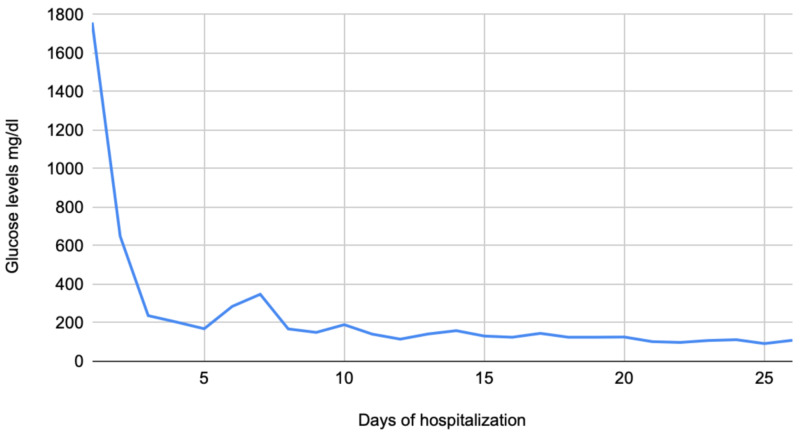
Glucose levels during hospitalization

A 36-year-old male with a past medical history of paranoid schizophrenia and bipolar disorder, on lithium 900 mg daily, risperidone 3 mg daily and quetiapine 300 mg daily presented to the emergency department (ED) with altered mental status causing multiple witnessed falls. The family reported no other past medical history and he was seen drinking two to three litters per day of carbonated soft drinks (Coke) for the past couple of days. On admission, he was drowsy with a Glasgow Coma Scale (GCS) of 10. There were coarse breath sounds on lung auscultation. His blood testing was significant for glucose of 1,756 mg/dl, hemoglobin glycosylated (HbA1c) 13.6%, white blood count 16.3 K/UL, sodium 152 mmol/L, creatinine 2.51 mg/dl and creatinine kinase 228 IU/L. Lithium levels were normal, and a urine drug screen was negative. CT scan of the head was unremarkable. He was initially admitted to the critical care unit for a hyperosmolar hyperglycemic state (HHS), and glucose levels were stabilized over the hospital course (Figure 1).

**Figure 2:**
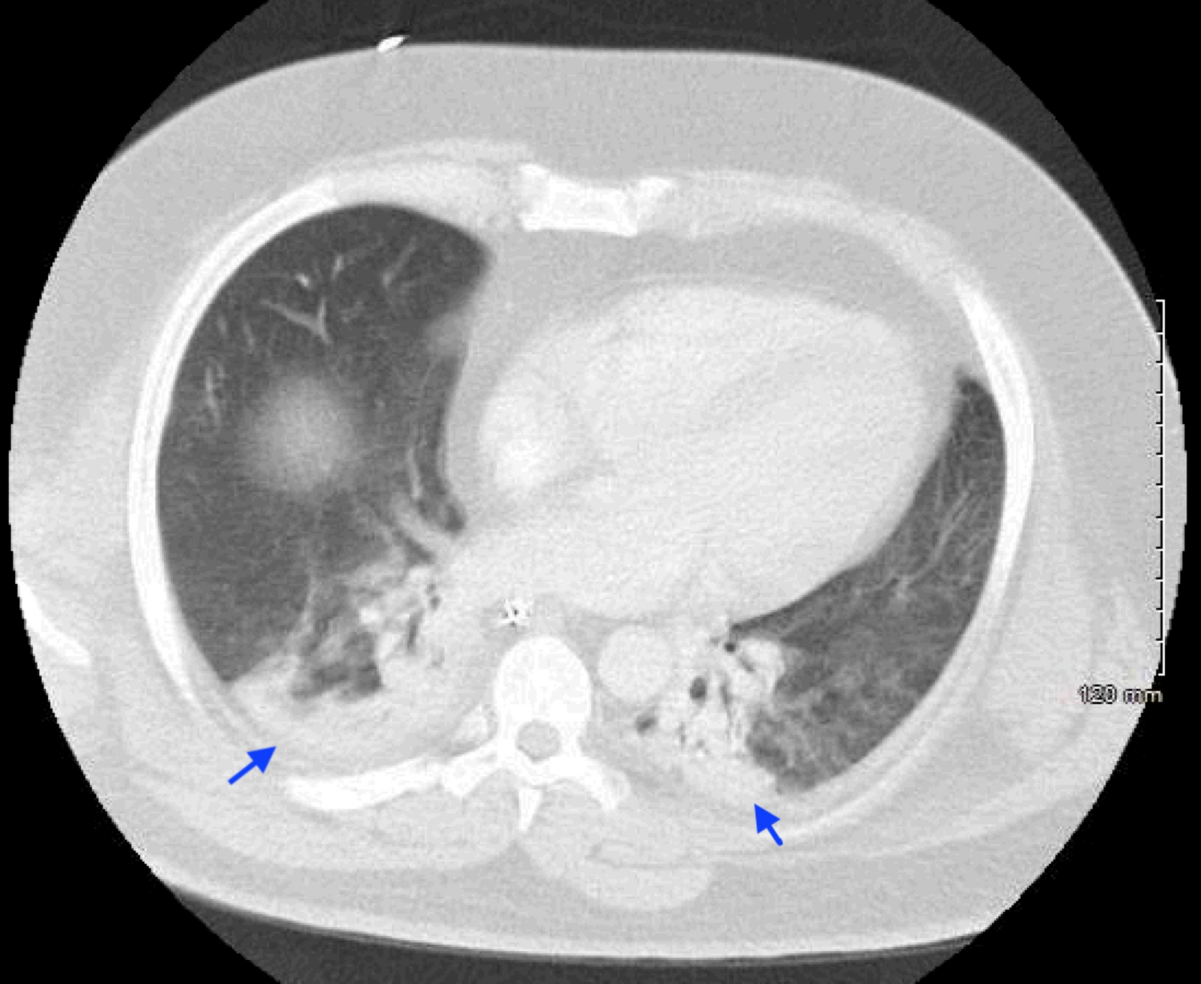
CT chest with bilateral lower lung consolidations

He was also found to have severe sepsis secondary to influenza type A and Klebsiella pneumoniae (Figure 2). Then, he developed neuroleptic malignant syndrome (NMS) with severe rhabdomyolysis and acute kidney injury requiring hemodialysis. After being hospitalized 26 days, he was transferred to a long term acute care (LTAC) facility and was discharged only on divalproex 500 mg twice daily and low-dose scale insulin (LDSSI) 1-5 units lispro as needed.

## Discussion

Patients with mental illness have limited access to the health system and are also unfortunately subjected to pharmacological side effects of psychiatric medications. This combination increases the risk of acquiring T2DM. A large meta-analysis on patients with severe mental illness revealed that 2.9% of the population had DM before treatment and post-treatment the incidence increased to 11.3% [3]. It is common for these patients to present with a hyperglycemic emergency due to the delay in diagnosing diabetes. Most of the patients in other studies had elevated HbA1C, suggesting undiagnosed T2DM at least several weeks before psychiatric patients presented to ED with complications [4]. Antipsychotics contribute to glucose intolerance through serotonin receptor (5HT2C) and histamine receptor (H1) antagonism in the hypothalamus resulting in increased appetite, weight gain, and insulin resistance [5]. In our case, the patient presented with an HbA1c of 13.6%, confirming undiagnosed DM. 

## Conclusions

This case highlights the importance of DM screening among patients on antipsychotic medications. These drugs cannot be stopped; therefore, preventive medicine plays an important role. Currently, there are no firm guidelines for metabolic screening in this high-risk patient population but at least they should be screened when they are started on antipsychotics.
